# Supplementary material for: Social expectations and government incentives in Malaysia’s COVID-19 vaccine uptake
Source: PLoS One. 2022 Sep 26;17(9):e0275010. doi: 10.1371/journal.pone.0275010 (PMC9512221; doi:10.1371/journal.pone.0275010)
Supplement: S1 Appendix — Survey questions used on Qualtrics. (PDF) [file pone.0275010.s001.pdf]

ASB Vaccine Survey

Start of Block: Select Language

Q1 Choose your Language

- O English (1)
- O Bahasa Melayu (2)
- O 中文 (3)

End of Block: Select Language

Start of Block: English - Base

Consent - English This survey was designed and developed by a research team at the Asia School of Business. It consists of 4 parts, which will not take more than 10 minutes to complete. You may stand a chance to win 10 cash prizes worth RM100 each in our lucky draw for participating. This survey is intended to understand vaccine hesitancy and whether social norms or government policies play roles in affecting one's decision to register for vaccination. By agreeing to participate in this study, you understand: The nature and scope of this research. That you voluntarily agree to participate in this research. That you can withdraw at any time without giving any reason. That any information collected is completely private and confidential. Only researchers will see your responses. Any reports or papers or publications written from data in this survey will NOT identify you. For further information about this research, please contact Fathin Rusliza at fathin.rusliza@asb.edu.my.
Once you move to the next page you won't be able to revisit previous questions. Please answer questions carefully.

- O I understand and I consent to participate in this survey. (1)

Page Break

Q1 The completion of this survey will qualify you to participate in a completion lucky draw for 10 cash prizes worth RM 100 each. Do you consent to provide us with your phone number and IC or passport number that you used for vaccine registration so that we may verify your vaccine registration status?

Yes I consent to provide my phone number and IC/Passport number (1)

I do not want to share this information. (2)

Display This Question:

If The completion of this survey will qualify you to participate in a completion lucky draw for 10 c... = Yes I consent to provide my phone number and IC/Passport number

Q135 Please provide your phone number and IC/Passport Number here.

O Phone Number (1) ____

O IC or Passport Number (2) ____

Q2 Where do you live?

- O Johor (1)
- O Kedah (2)
- O Kelantan (3)
- O Kuala Lumpur (4)
- O Melaka (5)
- O Negeri Sembilan (6)
- O Pahang (7)
- O Perak (8)
- O Perlis (9)
- O Pulau Pinang (10)
- O Sabah (11)
- O Sarawak (12)
- O Selangor (13)
- O Terengganu (14)
- O Putrajaya (15)
- O Wilayah Persekutuan Labuan (16)

Q3 What best describes your residential areas?

- O Village (1)
- O New village (2)
- O Town (3)
- O City (4)

Q4 How old are you?

____

Q5 Which religion do you most identify with?

- O Islam (1)
- O Hinduism (2)
- O Buddhism (3)
- O Taoism (4)
- O Christianity (5)
- O None (6)
- O Other (7) ____

Q6 What is your gender?

- O Male (1)
- O Female (2)
- O Prefer not to say (3)

Q7 What is the highest education level completed?

- O No formal education (1)
- O Primary school (2)
- O Secondary school (3)
- O Diploma (4)
- O Degree (5)
- O Master's degree or higher (6)

Q8 Below is an income scale on which 0 indicates the lowest and 10 the highest median monthly salaries and wages of employees in your country. As a reference, an individual's monthly salaries and wages in group 5 is RM2,442 in Malaysia. (Source: Department of Statistics Malaysia, 2020)

Where would you place your take home pay on this scale?

- O 0 (0)
- O 1 (1)
- O 2 (2)
- O 3 (3)
- O 4 (4)
- O 5 (5)
- O 6 (6)
- O 7 (7)
- O 8 (8)
- O 9 (9)
- O 10 (10)

Q9 Do you have the following apps on your phone? (Select all that apply.)

- [ ] MySejahtera (1)
- [ ] WhatsApp (2)
- [ ] Telegram (3)
- [ ] Facebook (4)
- [ ] WeChat (5)
- [ ] Twitter (6)
- [ ] Instagram (7)
- [ ] TikTok (8)

Q10 This is a list of 6 groups that have social and economic interactions with you. Provide the percentage (%) that you think and believe has been registered to be vaccinated within each group. (Select all that apply and answer).

	Not Applicable<br>0 10 20 30 40 50 60 70 80 90 100
People who live with you ()	
Family ()	
Friends ()	
Coworkers ()	
Neighbours ()	
Members of your religious group ()	

Q11 Do you believe that you and those in your social and economic circles benefit from vaccinations?

O Yes (1)

O No (2)

Q12
Do you believe that the majority of the individuals in the group(s) listed below are currently refusing the COVID-19 vaccine? (Select all that apply.)

▢ People who live with you (1)

▢ Your family members (that do not live with you) (2)

▢ Your friends (3)

▢ Your coworkers (4)

▢ Your neighbours (5)

▢ Everyone in my social and economic circles HAS NOT REFUSED to register and get vaccinated (6)

Page Break

Q13 Generally speaking, would you say that most people can be trusted or that you need to be very careful in dealing with people?

- O Most people can be trusted (1)
- O Need to be very careful in dealing with people (6)

Q14 How much do you trust people from the following groups?

	1 - Don't Trust At All (1)	2 - Do not Trust Very Much (2)	3 - Trust Somewhat (3)	4 - Trust Completely (4)	5 - Not Relevant to Me (5)
Family members (1)	o	o	o	o	o
Your neighbours (2)	o	o	o	o	o
Religious leaders (3)	o	o	o	o	o
Local council (4)	o	o	o	o	o
Your Member of Parliament or ADUN (5)	o	o	o	o	o
Ministry of Health (Kementerian Kesihatan Malaysia) (6)	o	o	o	o	o
JAKIM (Department of Islamic Development Malaysia) (7)	o	o	o	o	o
Police (8)	o	o	o	o	o
Medical doctors (9)	o	o	o	o	o
Local scientists (10)	o	o	o	o	o
Foreign scientists (11)	o	o	o	o	o

Display This Question:

If The completion of this survey will qualify you to participate in a completion lucky draw for 10 c... = Yes I consent to provide my phone number and IC/Passport number

Q15 If we have further follow-up questions for you regarding this survey, may we contact you via your phone number?

- O Yes (1)
- O No (2)

End of Block: English - Base

Start of Block: English Herd Immunity

Q16 The spread of COVID-19 can be stopped if most of us are vaccinated. This is called herd immunity, which can only be achieved if most people get the vaccine. With the vaccine, we can protect ourselves, our families, our communities and our country.

We will now ask some questions about herd immunity and vaccination. **Bear in mind there is no correct or wrong answer. We are interested to know what YOU THINK and YOUR PERCEPTION.**

Q17 Please estimate the percentage of COVID-19 vaccination registration rate in your state now.

	0 10 20 30 40 50 60 70 80 90 100
Your Response ()	

Q18 Please estimate the percentage of the population in your state that is refusing to register for the COVID-19 vaccination.

0 10 20 30 40 50 60 70 80 90 100

Your Response ()	

Q19 Please estimate the percentage of the population in your state that needs to be vaccinated to achieve herd immunity.

	0 10 20 30 40 50 60 70 80 90 100
Your Response ()	

Q20 Please estimate the percentage of the population in your state that supports government action to make vaccination mandatory and punish those that refuse vaccination.

	0 10 20 30 40 50 60 70 80 90 100
Your Response ()	

Q21 Please estimate the percentage of the population in your state that supports government action to make COVID-19 vaccine accessible and to provide more incentives to get vaccinated.

	0 10 20 30 40 50 60 70 80 90 100
Your Response ()	

Q22 Have you registered for COVID-19 vaccine? Please also select YES if you’ve already been vaccinated.

O Yes (1)

O No (2)

End of Block: English Herd Immunity

Start of Block: English Lucky Draw

Control Group Thank you for your time. We will be in contact if you are one of the winners of the completion lucky draw.

Treatment Group We are offering an additional lucky draw of one prize of RM 500 to incentivize people to sign up for the vaccine. To qualify, you need to sign up on https://www.vaksincovid.gov.my/ or MySejahtera by midnight, Monday 5th of July 2021. You don't need to send us any additional information. We will check your vaccination status and contact the winner.

End of Block: English Lucky Draw

Start of Block: ENGLISH ; Section A: I have registered to get the vaccine

Q29 Suppose that there are individuals from your social and economic circles who refuse to be vaccinated. Knowing this, what are your responses if you have to deal with them once the MCO restrictions have loosened and **you've already been vaccinated**? Pick one.

- O Maintain social distancing and mask (1)
- O Engage in extra effort to avoid these individuals like eating alone OR avoid sharing a bathroom with them OR avoid sending my kids to schools/kindergarten if they have kids in the same place (2)
- O I feel confident to not wear a mask around them (3)

Q30 Have you been vaccinated with at least one dose already?

- Yes: in February (1)
- Yes: in March (4)
- Yes, in April (5)
- Yes, in May (6)
- Yes, in June (7)
- No (2)

Q31 When did you first register for the vaccination program?

- February 2021 (vaccine registration on MySejahtera started on February 22nd) (1)
- March 2021 (2)
- April 2021 (3)
- May 2021 (opt-in for AstraZeneca in Klang Valley started on May 2nd) (4)
- June 2021 (5)

Q32 How did you sign up for the vaccination program?

- O Directly on my phone through MySejahtera (1)
- O Directly on https://www.vaksincovid.gov.my/ website (2)
- O Done by other family members on MySejahtera or website (3)
- O Walk-in at the nearest Klinik Kesihatan or Pusat Kesihatan Daerah (4)
- O Put my name and details in a list compiled by employer / JKKK Kampung / trade or professional association or wakil khidmat masyarakat ADUN or MP (5)
- O Others (6) ____

Q33 Given current policies, do you believe that the nation is on track to achieving herd immunity?

- O Yes (1)
- O No (2)

Q34 When do you expect the benefit of herd immunity can be felt?

- O By the end of this year 2021 (1)
- O Early next year (Jan to March 2022) (2)
- O Mid of next year (May to June 2022) (3)
- O Any period after June 2022 (4)

Q35 Did you sign up immediately for the vaccine?

- Yes (1)
- No (2)

Display This Question:

If Did you sign up immediately for the vaccine? = Yes

Q36 Why did you sign up immediately? (Please select all that apply.)

- ▢ Friends and family encouraged me to take the vaccine (1)
- ▢ I want to develop immunity towards COVID-19 (2)
- ▢ I live with high-risk individuals (3)
- ▢ I trust politicians (4)
- ▢ I trust Kementerian Kesihatan Malaysia (Ministry of Health) (5)
- ▢ It is required for my work. (6)
- ▢ The government offered specific vaccines that I want to take (7)
- ▢ I was worried about the increase in COVID-19 cases and deaths (8)
- ▢ I received incentives to sign up (9)
- ▢ I do not want to be punished or fined (10)
- ▢ I want to be safe when I meet people from outside my household (11)
- ▢ Others (12) ____

Display This Question:

If Did you sign up immediately for the vaccine? = No

Q37 Why didn't you immediately sign up? (Please select all that apply.)

- ☐ I was worried about the long-term effects of the vaccine (1)
- ☐ I was worried about the immediate side-effects from the vaccine (2)
- ☐ I was unsure that the vaccine will work (3)
- ☐ I was doubtful of the ingredients used to produce the vaccine (4)
- ☐ I already got COVID-19 (5)
- ☐ My family members did not let me register for the vaccine (6)
- ☐ I did not think there will be an available vaccine for me so I waited (7)
- ☐ I wanted to see more people get vaccinated before I registered (8)
- ☐ The authorities could not guarantee me the type of vaccine that I want (9)
- ☐ I did not trust politicians (10)
- ☐ I did not trust Kementerian Kesihatan Malaysia (KKM) (11)
- ☐ I was afraid to leave my house to get the vaccine (12)
- ☐ I didn't understand how to register or it is too hard (13)
- ☐ I had no time (14)
- ☐ Others (15) ____

Display This Question:

If Did you sign up immediately for the vaccine? = No

Q38 What made you finally sign up? (Please select all that apply.)

- ☐ Friends and family encouraged me to take the vaccine (1)
- ☐ I want to develop immunity towards COVID-19 (2)
- ☐ I live with high-risk individuals (3)
- ☐ I trust politicians (4)
- ☐ I trust Kementerian Kesihatan Malaysia (Ministry of Health) (5)
- ☐ It is required for my work (6)
- ☐ The government offered specific vaccines that I want to take (7)
- ☐ I was worried about the increase in COVID-19 cases and deaths (8)
- ☐ I received incentives to sign up (9)
- ☐ I do not want to be punished or fined (10)
- ☐ I want to be safe when I meet people from outside my household (11)
- ☐ Others (12) ____

End of Block: ENGLISH ; Section A: I have registered to get the vaccine

---

Start of Block: English - Section B

Q29 Why have you not registered for the vaccine? (Please select all that apply.)

- ☐ I am worried about the long-term effects of the vaccine (16)
- ☐ I am worried about the immediate side-effects from the vaccine (17)
- ☐ I am unsure that the vaccine will work (18)
- ☐ I am doubtful of the ingredients used to produce the vaccine (19)
- ☐ I already got COVID-19 (20)
- ☐ My family members do not let me register for the vaccine (21)
- ☐ I do not think there will be an available vaccine for me so I want to wait (22)
- ☐ I want to see more people get vaccinated before I registered (23)
- ☐ The authorities cannot guarantee me the type of vaccine that I want (24)
- ☐ I do not trust politicians (25)
- ☐ I do not trust Kementerian Kesihatan Malaysia (KKM) (26)
- ☐ I am afraid to leave my house to get the vaccine (27)
- ☐ I do not understand how to register or it is too hard (28)
- ☐ I have no time (29)
- ☐ Others (30) ____

Q30
If you could be guaranteed the vaccine of your choice at the time and location of your choice in the next month, would you register for the vaccine today?

- O Yes (1)
- O No (2)

End of Block: English - Section B

Start of Block: English - Section Vignettes

Vignette 1
Achieving herd immunity is a goal of many governments in the world. Imagine that somebody like you lives in Malaysia and hasn't registered for the vaccine. In this scenario, most residents in Malaysia have refused to register for the vaccine. The government is providing an incentive to get registered and vaccinated by conducting a nationwide lucky draw to win RM 1 million.

How likely is this person to register for vaccination in this situation? (1)	O Extremely Unlikely (1)	O Unlikely (2)	O Neutral (3)	O Likely (4)	O Extremely Likely (5)
How likely is it that this person actually gets vaccinated in this situation? (2)	O Extremely Unlikely (1)	O Unlikely (2)	O Neutral (3)	O Likely (4)	O Extremely Likely (5)
Do you think this person should get vaccinated in this situation? (3)	O Strongly Disagree (1)	O Disagree (2)	O Neutral (3)	O Agree (4)	O Strongly Agree (5)

Vignette 2 Achieving herd immunity is a goal of many governments in the world. Imagine that somebody like you lives in Malaysia and hasn't registered for the vaccine. In this scenario, most residents in Malaysia have refused to register for the vaccine. The government is punishing residents by requiring that non-vaccinated residents must pay for PCR Covid tests every 14 days in order to work in person.

How likely is this person to register for vaccination in this situation? (1)	O Extremely Unlikely (1)	O Unlikely (2)	O Neutral (3)	O Likely (4)	O Extremely Likely (5)
How likely is it that this person actually gets vaccinated in this situation? (2)	O Extremely Unlikely (1)	O Unlikely (2)	O Neutral (3)	O Likely (4)	O Extremely Likely (5)
Do you think this person should get vaccinated in this situation? (3)	O Strongly Disagree (1)	O Disagree (2)	O Neutral (3)	O Agree (4)	O Strongly Agree (5)

Vignette 3
Achieving herd immunity is a goal of many governments in the world. Imagine that somebody like you lives in Malaysia and hasn't registered for the vaccine. In this scenario, most residents in Malaysia have registered for the vaccine. The government is providing an incentive to get registered and vaccinated by conducting a nationwide lucky draw to win RM 1 million.

How likely is this person to register for vaccination in this situation? (1)	O Extremely Unlikely (1)	O Unlikely (2)	O Neutral (3)	O Likely (4)	O Extremely Likely (5)
How likely is it that this person actually gets vaccinated in this situation? (2)	O Extremely Unlikely (1)	O Unlikely (2)	O Neutral (3)	O Likely (4)	O Extremely Likely (5)
Do you think this person should get vaccinated in this situation? (3)	O Strongly Disagree (1)	O Disagree (2)	O Neutral (3)	O Agree (4)	O Strongly Agree (5)

Vignette 4 Achieving herd immunity is a goal of many governments in the world. Imagine that somebody like you lives in Malaysia and hasn’t registered for the vaccine. In this scenario, most residents in Malaysia have registered for the vaccine. The government is punishing residents by requiring that non-vaccinated residents must pay for PCR Covid tests every 14 days in order to work in person.

How likely is this person to register for vaccination in this situation? (1)	O Extremely Unlikely (1)	O Unlikely (2)	O Neutral (3)	O Likely (4)	O Extremely Likely (5)
How likely is it that this person actually gets vaccinated in this situation? (2)	O Extremely Unlikely (1)	O Unlikely (2)	O Neutral (3)	O Likely (4)	O Extremely Likely (5)
Do you think this person should get vaccinated in this situation? (3)	O Strongly Disagree (1)	O Disagree (2)	O Neutral (3)	O Agree (4)	O Strongly Agree (5)

End of Block: English - Section Vignettes

Start of Block: Malay - Base

Q49 **PERSETUJUAN MENYERTAI KAJI SELIDIK** Kaji selidik ini dilaksanakan oleh pasukan penyelidik daripada Asia School of Business, Malaysia untuk mengkaji berkenaan keraguan terhadap vaksin dan memahami bagaimana norma sosial atau dasar negara mempengaruhi keputusan seseorang untuk mendaftar vaksinasi. Kaji selidik ini mengandungi 4 bahagian dan dianggarkan tidak akan mengambil masa lebih daripada 15 minit untuk menjawab. Dengan menyertai kaji selidik ini, anda berpeluang untuk memenangi 10 hadiah wang tunai cabutan bertuah bernilai RM100. Dengan bersetuju untuk mengambil bahagian dalam kaji selidik ini, anda memahami: Skop kaji selidik ini Bahawa anda secara sukarela bersetuju untuk
