# Supplementary material for: Social expectations and government incentives in Malaysia’s COVID-19 vaccine uptake
Source: PLoS One. 2022 Sep 26;17(9):e0275010. doi: 10.1371/journal.pone.0275010 (PMC9512221; doi:10.1371/journal.pone.0275010)
Supplement: S2 Appendix — (PDF) [file pone.0275010.s002.pdf]

S2 Appendix. Supplementary Analyses

A. Further analysis of empirical expectations and registration outcomes

Table S1.Correlation of vaccine registration with expectations on population's refusal and registration

	Registered for vaccination	
	(1)	(2)
Percentage of state's population refuse vaccination	-0.00125***	
	(0.000427)	
Percentage of state's population registered		0.000520*
		(0.000294)
Observations	1294	1297

Standard errors in parentheses

* $p < 0.10$, ** $p < 0.05$, *** $p < 0.01$

Table S2. Correlation of belief in vaccine benefits population's refusal and registration

	Belief in vaccination benefits	
	(1)	(2)
Percentage of state's population refuse vaccination	-0.00112***	
	(0.000251)	
Percentage of state's population registered		0.000605***
		(0.000172)
Observations	1294	1297

Standard errors in parentheses

* $p < 0.10$, ** $p < 0.05$, *** $p < 0.01$

Table S3. Effect of incentives and expectations on hypothetical vaccination behavior

	Will register	Will get vaccinated	Should get vaccinated
	(1)	(2)	(3)
HP	0.00345	-0.0362	0.0661*
	(0.0377)	(0.0391)	(0.0351)
LOT	-0.0776**	-0.138***	-0.155***
	(0.0371)	(0.0385)	(0.0346)
HP X LOT	0.0467	0.0700	-0.0456
	(0.0518)	(0.0538)	(0.0483)
Observations	1244	1243	1244

Standard errors in parentheses

* $p < 0.10$, ** $p < 0.05$, *** $p < 0.01$

Tables S1 and S2 suggest that empirical expectations on others' vaccination decisions have a small but positive effect on behavior and norms: actual registration and belief that vaccines benefit one's community–real-world analogues for the hypothetical will register and should register vignette questions, respectively–show mostly significant positive correlations with perceived state level registration and negative correlations with perceived state level vaccine refusal. However, the magnitudes of these effects are relatively small–for comparison to the vignette manipulations, suppose high registration is interpreted as 50% higher than low registration, or reflects a similar difference in refusal percentage, and that the effect of a 1% increase in perceived registration or refusal rate on the "will register" and "should register" outcomes is given by the coefficients in Tables S1 and S2. Then the increased probabilities of those outcomes range from 0.025 to 0.06, comparable to the coefficients on HP and HP x LOT seen in Table S3 and small relative to the standard errors in that regression. Effects of this size will not be significant in the vignette experiment, even if they're real.

Table S4. Variation explained by state fixed effects for various state-level social expectations

	Percentage of state's population			
	refuse vaccine	support for incentive	support for punishment	registered
	(1)	(2)	(3)	(4)
State fixed effects	Yes	Yes	Yes	Yes
Observations	1294	1295	1294	1297
R^2	0.077	0.022	0.021	0.041

Table S4 shows there is very little variation in these expectations is explained by the respondent's state of residdent. Variation in these expectations are almost entirely due to individual idiosyncrasies in social expectations. State-level omitted variables cannot explain their relationship with vignette outcomes.

Supplementary analysis on trust results

Table S5. Effect of incentives, norms, and beliefs on hypothetical vaccination behavior and trust towards Ministry of Health

	(1)<br>Will Register	(2)<br>Should get vaccinated
LOT	0.00887	-0.145***
	(0.0571)	(0.0525)
Trust MOH	0.155***	0.124***
	(0.0465)	(0.0427)
LOT X Trust MOH	-0.0868	-0.0484
	(0.0640)	(0.0589)
Registered for vaccination	0.163**	0.270***
	(0.0696)	(0.0640)
HH Expectation	0.00000831	0.000807*
	(0.000473)	(0.000435)
Live Alone	-0.0497	-0.0225
	(0.0550)	(0.0505)
Support Intervention	0.00114***	0.00114***
	(0.000344)	(0.000316)
N	1221	1221

Support Intervention calculate by averaging expected state's population share that support rewards with the state's population share the support punishment in the respondent's state.Standard errors in parentheses. Significant levels: * $p < 0.10$, ** $p < 0.05$, *** $p < 0.01$

Table S6. Expected support for government policies, perceived refusal and trust towards Ministry of Health

	Percentage of state's population	
	support for incentive	support for punishment
State refusal rate	-0.289***	-0.132***
	(0.0350)	(0.0430)
Trust MOH	1.794	5.866***
	(1.456)	(1.788)
State fixed effects	Yes	Yes
Observations	1273	1272

Standard errors in parentheses

* $p < 0.10$, ** $p < 0.05$, *** $p < 0.01$

The "generalized trust" could influences vignette outcomes via an additional channel: distrust in the protagonist. To test this possibility, in Table S5 we re-estimate our main regression model for registration and norms.All results for vaccination are extremely similar to registration, so we drop further analysis of that outcome, replacing generalized trust with trust in the MOH. If distrust towards the protagonist is true, we should see significantly lower belief that the protagonist should get vaccinated for low MOH trust individuals, and in fact this is what Table S5 shows. Further, insignificant mediating effects of MOH trust on the behavioral effect of punishments versus rewards suggest the distrust of authority mechanism could be the mediating effects on behavior also be partially driven by distrust of the protagonist.

Furthermore lower government legitimacy leads to a relatively higher efficacy for reward interventions, Table S6 shows that perceived percentage of state's population refusal rate is strongly negatively correlated with perceived support for interventions, and has a stronger association with support for incentives than support for punishment. Almost all believe that incentives are more widely supported than punish-

ments, but belief in support for incentives drops more sharply than for punishments for those who perceive high refusal rates.
