# Supplementary material for: Social expectations and government incentives in Malaysia’s COVID-19 vaccine uptake
Source: PLoS One. 2022 Sep 26;17(9):e0275010. doi: 10.1371/journal.pone.0275010 (PMC9512221; doi:10.1371/journal.pone.0275010)
Supplement: Attachment — Submitted filename: Response to Reviewers.pdf [file pone.0275010.s003.pdf]

UNIVERSITI KEBANGSAAN MALAYSIA
The National University of Malaysia

INSTITUT KAJIAN MALAYSIA DAN ANTARABANGSA • *INSTITUTE OF MALAYSIAN AND INTERNATIONAL STUDIES*

5 September 2022

Dear Srikanth Umakanthan,
Academic Editor of PLOS ONE

We thank you for your constructive comments in helping us improve our manuscript. We have made all changes requested and responded to all comments. We have also sent the manuscript to a trusted proof-reader in order to spot any errors and checks the clarity of the study. With this letter, we also submitted the revised manuscript in 2 forms, i) marked-up copy with changes suggested by the reviewers, and ii) unmarked copy. Our comments are in blue. We hope to hear from you soon.

Your comment:
The discussion requires more additional statements with regards to the existing literature search. Include the following references and citations reflecting the COVID-19 updates to strengthen the manuscript:
- Origin and transmission (use reference and cite: doi:10.1136/postgradmedj-2020-138234)
- To mention in brief about vaccines (use reference and cite: “doi: 10.1136/postgradmedj-2021-141365.
AND doi:10.1136/postgradmedj-2021-140654”)
- Definition of vaccine resistance and hesitance (use reference and cite: doi:10.3390/vaccines9101064).
- Compare the global vaccine status and relate it with the current vaccine status (refer and cite: doi: 10.3389/fpubh.2022.844333)

Our response:
Thank you for your suggestions. We have included relevant statements that touch issues mentioned above to improve our manuscript. The additional statements relevant to your suggestions are underlined with a short note on the margin. The suggested and related references added in the marked-up revised version are the References in blue font.

We noticed that the list of tables in the Supplementary Analyses are repeated under Tables S1 to S6 in the originally submitted manuscript. So to avoid redundancy of tables submitted we have deleted the list. Readers should be able to access the tables in 1 PDF file labelled ‘S2 Appendix, Supplementary Analyses’.

Thank you very much for considering our manuscript. Please let me know if you have any questions about the manuscript – I can be reached at the izzatina.aziz@ukm.edu.my.

Sincerely,

NOR IZZATINA ABDUL AZIZ
Junior Research Fellow

CERTIFIED TO ISO 9001:2015 CERT. NO. QMS 01100

INSTITUT KAJIAN MALAYSIA DAN ANTARABANGSA
Universiti Kebangsaan Malaysia, 43600 UKM Bangi, Selangor Darul Ehsan Malaysia
Tel.: +603-8921 4169 Faks: +603-8926 1022 E-mel: pghikmas@ukm.edu.my Web: www.ukm.my/ikmas

Mengilham Harapan, Mencipta Masa Depan • *Inspiring Futures, Nurturing Possibilities* www.ukm.my
